# Adherence Rate, Barriers to Attend, Safety, and Overall Experience of a Remote Physical Exercise Program During the COVID-19 Pandemic for Individuals After Stroke

**DOI:** 10.3389/fpsyg.2021.647883

**Published:** 2021-07-09

**Authors:** Camila Torriani-Pasin, Gisele Carla dos Santos Palma, Marina Portugal Makhoul, Beatriz de Araujo Antonio, Audrea R. Ferro Lara, Thaina Alves da Silva, Marcelo Figueiredo Caldeira, Ricardo Pereira Alcantaro Júnior, Vitoria Leite Domingues, Tatiana Beline de Freitas, Luis Mochizuki

**Affiliations:** ^1^Laboratory of Motor Behavior, School of Physical Education and Sport, University of São Paulo, São Paulo, Brazil; ^2^School of Arts, Sciences and Humanities, University of São Paulo, São Paulo, Brazil

**Keywords:** stroke, telemonitoring, barriers, COVID-19, physical activity, telehealth, social isolation, physical exercies

## Abstract

**Introduction:** The actions taken by the government to deal with the consequences of the coronavirus diseases 2019 (COVID-19) pandemic caused different levels of restriction on the mobility of the population. The need to continue offering physical exercise to individuals after stroke became an emergency. However, these individuals may have barriers to adhere to the programs delivered remotely. There is a lack of evidence related to adherence, attendance, safety, and satisfaction of remote exercise programs for this population.

**Objective:** The aim was to evaluate adherence and barriers to attend a remote physical exercise program for individuals after stroke. We aimed (a) to identify adherence and attendance rate of the remote physical exercise program (i.e., number of participants engaged, number of sessions attended, and exercise time in remote program); (b) to identify the safety of a remote physical exercise program (i.e., falls, pain, or dizziness when performing the exercises, fear, or insecurity); and (c) to identify the overall experience to participate in a remote program.

**Materials and methods:** This is a longitudinal study, including 36 stroke survivors who already attended a face-to-face physical exercise program prior to the COVID-19 pandemic. The remote physical exercise program included sessions for 2 days/week for a duration of 22 weeks, with a total of 44 sessions, which were delivered asynchrony *via* recorded video sessions. As outcome measures, we performed two questionnaires (via weekly telephone calls) to identify attendance, barriers, safety, and overall experience related to the program.

**Results:** The adherence rate was 86 (9%). The attendance rate was 19, with a total of 8 sessions (108.3 min/week). The main barriers for lower attendance rate were as follows: lack of motor skills and physical fitness to workout in 80 reports (20.6%), followed by no exercise companion in 44 reports (11.3%). The remote physical program has been shown to be safe, and the overall experience was positive from the perspectives of the participants and the family members.

**Conclusion:** Although the adherence rate was high, the attendance rate was low on the remote physical exercise program. The main barriers to attending the program remotely reflect the need of tailoring individually an asynchrony mode of delivering the sessions to individuals after stroke. Our results also indicate how the COVID-19 impacted the health conditions of stroke survivors. The program was safe, and the overall experience indicated a change in the mental, physical, and social health of individuals after stroke and their family members.

## Introduction

Social distancing was applied to mitigate the effects of the pandemic caused by the severe acute respiratory syndrome coronavirus 2 (SARS-CoV-2) [coronavirus disease 2019 (COVID-19)], leading to mobility restrictions and suspending face-to-face services. Physical exercise facilities (e.g., gyms and public parks) were closed, constraining the physical activity for physical conditioning, therapeutic exercise, or leisure purposes. In particular, physical activity programs for individuals with chronic health conditions were suspended or were dramatically reduced. To overcome such limitations, alternatives based on remote-supervised activities emerged (Hosseiniravandi et al., 2020).

Since the late 1960s, the development of Internet protocols has created opportunities for teleservices, such as monitoring and supervision. At present, remote sensing, nanotechnology, and the Internet of Things have potentiated the emergence of mHealth or health services provided at a distance (McCue et al., 2010; Linder et al., 2015). Remote physical exercise programs (e.g., telerehabilitation, telemonitoring, or home-based rehabilitation) can offer guided exercises with remote monitoring to people with musculoskeletal and neurological disorders (Hosseiniravandi et al., 2020), who are isolated by social distance. Such remote monitoring allows health professionals to adapt the exercises and physical activities according to the needs of the individuals with neurological diseases (Laver et al., 2020), facilitates access to health services, and improves equity, thus reducing the costs of the rehabilitation programs (Chen et al., 2020; Ghorbel et al., 2020; Han et al., 2020; PUi Kei et al., 2020; Salawu et al., 2020; Zedda et al., 2020).

Stroke is a neurological condition that can cause impairments in functions and structures, limitation of activity, and restraint of participation. Thus, the practice of physical exercises is an approach that presents benefits in all affected areas (Saunders et al., 2020). In the current scenario, remote physical exercises were the only way to keep them active. Appleby et al. (2019) identified that a remote physical exercise program has moderate evidence to improve motor function, independence in activities of daily living (ADLs), satisfaction, and quality of life for individuals after stroke.

Laver et al. (2020) conducted a systematic review to compare telerehabilitation programs, face-to-face interventions, and usual care for stroke survivors, thus, less evidenced studies suggest that remote and face-to-face rehabilitation programs have similar effects on ADLs, balance, and upper limb function, while moderately evidenced studies suggest that remote programs and usual care (or no care) have similar effects on ADL independence, quality of life, and depression. Only two studies (Laver et al., 2020) reported adverse effects during the remote intervention.

Still, this review (Laver et al., 2020) raises some important points to be addressed in future studies, in particular, there is the importance of knowing whether study participants are satisfied with the program, and additionally, which participants could benefit more from this mode of delivery. At the same time, barriers to engaging in remote physical exercise programs during such a pandemic are not clearly stated. Individuals with neurological disorders have barriers to attend a face-to-face program, e.g., time restrictions; resource limitations; geographical isolation; compliance with rehabilitation; fear of falling and more severe motor restrictions; and expectation of low results (Dobkin, 2016; Afshari et al., 2017; Appleby et al., 2019). Would these same barriers affect similarly the adherence and attendance on a remote exercise program?

To answer this question, this study aims to evaluate barriers to adhere and attend remote physical exercise programs for individuals after stroke. Specifically, we aim (a) to measure the adherence rate and attendance to a remote physical exercise program (i.e., number of participants engaged, number of sessions attended, and exercise time); (b) to describe how safe a remote physical exercise program is (i.e., falls, pain, or dizziness when performing the exercises, fear, or insecurity); and (c) to measure the overall experience to participate in a remote physical exercise program for individuals after stroke and their family members.

## Materials and Methods

This is a phase-I clinical trial, and the CONSORT checklist is used. It was developed at the Motor Behavior Laboratory of the School of Physical Education and Sport of the University of São Paulo, São Paulo, Brazil. We have the approval of the Human Ethics Committee (# 4.119.009; CAAE No. 32005420.4.0000.5391; plataformabrasil.saude.gov.br). All individuals accepted to participate and provided their written informed consent.

### Participants

Individuals with a stroke diagnosis who attended the face-to-face community rehabilitation program at the School of Physical Education and Sport, University of São Paulo, São Paulo, Brazil earlier were invited to join this study after the COVID-19 pandemic had started. The inclusion criteria were as follows: confirmed diagnosis of stroke by image or medical report; either types of stroke (ischemic and hemorrhagic) in chronic phase (Bernhardt et al., 2017); above 18 years old; no orthopedic, other neurological, and cardiac risk factors to exercise; and Montreal Cognitive Assessment (MoCA) score higher than 14 [no dementia (Phannarus et al., 2020); walking speed ≥0.4 m/s (home walker) (Fulk et al., 2017); and perform the face-to-face community rehabilitation program for at least 6 months]. The exclusion criteria were to show a cardiovascular or respiratory condition impairing performance training and/or health safety during exercise. Individuals who missed sessions were not excluded because we aimed to identify the reasons and barriers for attendance. Therefore, the permanence of an individual in the study even without carrying out the activities becomes important. In terms of definition, the adherence rate was described as the relative frequency of individuals who accepted to engage in the remote exercise program (Ellis et al., 2019; Landers and Ellis, 2020).

### Characterization of Participants

Demographic data (e.g., sex, age, schooling, time since diagnosis, and known comorbidities before social isolation) of the participants were recorded previously in January and February, during the face-to-face physical exercise program, before the start of the COVID-19 pandemic in Brazil. Physical information from each participant, as a prerequisite to engaging in the face-to-face program, was obtained. The activity-specific balance confidence scale (ABC) (Branco, 2010) and the Mini-Balance Evaluation Systems test (MiniBESTest) (Bambirra et al., 2015) were used to characterize the perception of balance and balance itself; the 6-minute walk test (6 MWT) (ATS, 2002) was used to characterize aerobic capacity; the 10-meters walk test (10 m) (Tyson and Connell, 2009) was used to characterize the gait speed; the Timed Up and Go (TUG) (Richardson, 1991) test was used to characterize mobility; the MoCA was used to characterize cognitive deficit (Nasreddine et al., 2005); and the Stroke Impact Scale (SIS) was used to characterize the quality of life (Carod-Artal et al., 2008; Branco, 2010).

### Remote Exercise Protocol Development

An exercise protocol was developed for this remote program maintaining the same objective as the face-to-face program: to reduce physical inactivity, to increase physical function, such as aerobic capacity, muscle resistance, mobility, balance, and gait, and to improve balance confidence and cognition (Billinger et al., 2015). All activities follow the recommendations for physical exercise training for stroke survivors (Billinger et al., 2014; Sarfo et al., 2018; Saunders et al., 2020). Every Monday and Wednesday, a full session of videotaped activities was sent *via* mobile phone and email to all participants. The videos contained different exercises each day. Initially, the instruction on how to perform the exercise was given, such as execution time, perceived effort (intensity), the number of series, and repetitions given by the trainer. All exercises had different levels of intensity and complexity so that all participants were able to perform the exercises and adapt them to their own level. Table 1 shows the content of the videos with their respective aims, description of the activities, practice duration, and video duration. Participants were encouraged to do the exercise sessions on different, non-consecutive days. Required exercise equipment were household-like tools (e.g., broomstick, chair, cushion, and bottles of water or sand).

**Table 1:** Description of the content of the video sessions.

**Aim**	**Description**	**Practice duration**	**Video duration**
Warm-up	Low-intensity exercises with dual tasks, cognition, manual skills, and balance.	10 min	1–3 min
Aerobic	Low to moderate cyclic and rhythmic exercises with large muscle groups demanding the cardiorespiratory system.	12–15 min (two repetitions)	6 min
Resistance training	Dynamic and isometric exercise for trunk, lower, and upper limb. The prescription was three series between 15 and 20 repetitions depending on the muscles.	15 min	5 min
Coll down	To decrease the heart rate and blood pressure. Breathing and stretching exercises.	2 min	1–2 min

The attendance rate was described by the number of attended sessions based on 48 sessions in total. Additionally, the accumulated time (in min) per week of performed exercise was also considered. Furthermore, we aimed to describe the higher and lower attendance frequencies, including the participants who attended more than 80% and <20% of the sessions.

The working team for this remote exercise program was composed of a team leader (faculty member), a physical education instructor (faculty member), a health instructor team (four graduate students and an in-charge to supervise the intern team), and an intern team (20 undergraduate students). The entire team was trained and familiarized with how to apply the questionnaires. The intern team was in charge of weekly calls (voice or video calls) with each participant, where it was asked how hard it was to perform the proposed activities. Although every participant had the same videos, those weekly calls were designed to adapt the sessions to personal limitations and needs.

### Monitoring Procedures

A digital consent term copy was sent via mobile phone and email to all individuals who were interested in joining this study. The research team called the potential participants to explain all the procedures to them and their family members or their caregivers. Then, those who agreed to participate replied with the informed consent option checked.

Every week, the researcher team called each participant to talk about the barriers, how often he/she exercised, and how hard it was to exercise. Through a questionnaire using mostly yes-or-no answers, this tool assessed the perceived barriers (i.e., environmental, pandemic, and health condition related). Safety was evaluated based on adverse sensations (i.e., pain, dizziness, and nausea) during the session, fear, or insecurity to exercise, and adverse events such as falls. Participants also expressed their reasons not to do the remote physical exercise program in an open question. The perceived barriers were based on all the comments about how hard it was to exercise. These barriers were clustered within domains and counted.

### Developing Questionnaire: Overall Experience

**Figure 1:**
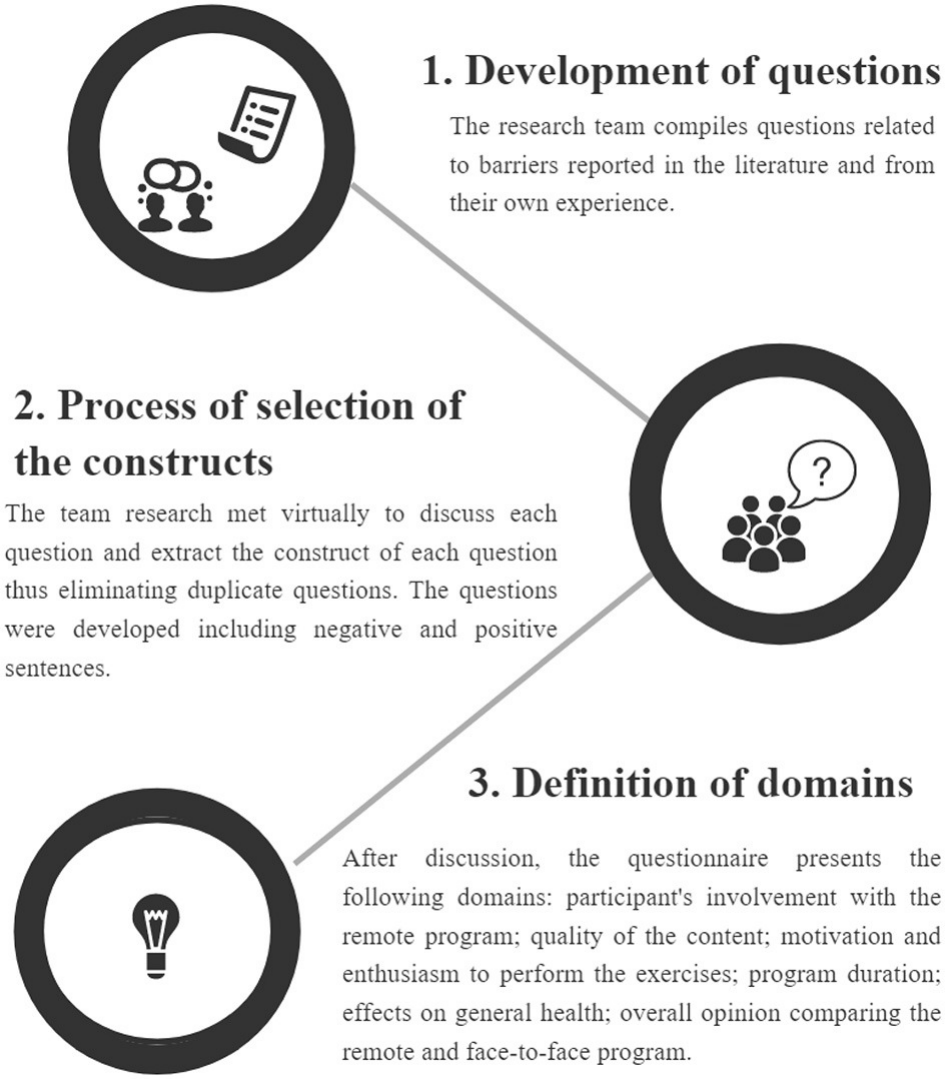
The development process of the overall experience questionnaire.

This questionnaire was developed in three steps (Figure 1). In step 1, questions were proposed. In step 2, writing and meaning were discussed in team meetings using a word cluster (Mentimeter, www.mentimeter.com) (Interactive Presentation Software–Mentimeter, 2020). Each research team member gave three words to identify each question, and the most cited words were used to define the construct of the questions. In step 3, constructs were clustered into domains. Table 2 depicts each question, construct, and domain. No validated questionnaire was found to assess barriers to remote exercises, and for this reason, this one was created by the authors. Given the urgent need to understand the limitations of physical exercise during the pandemic, it was not possible to validate the instrument satisfactorily.

**Table 2:** Constructs, subdomains, and definitions of the overall experience questionnaire.

**Construct**	**Domain**	**Questions**	**Definition**
Personnel	Satisfaction concerning the professional team	(2) The program made me feel safer in activities at home, such as walking. (3) The program interfered negatively with my mood and general health.	These questions are related to safety and health conditions while performing remote physical exercises.
Communication	Connectivity and interpersonal relationships between participants and team members	(7) During the online exercise program, I had easy communication with the professional team. (10) During the online exercise program, I had difficulties connecting with technology and I had problems with my internet connection.	These questions are related to the individual's perception of connectivity and communication with team members.
Program	Satisfaction concerning the program	(1) The format of this online program allowed me to participate and get involved satisfactorily. (4) I was not able to carry out all the activities and exercises prescribed to me. (5) I liked the online program and I think it could replace the face-to-face program. (8) During sessions, the professional showed that he understood my difficulties and managed to feedback on my performance, and provide the needed support to me.	These questions are intended to evaluate satisfaction, involvement, understanding of the exercises, the feasibility of performing exercises, and empathy.
Social support	Social interaction and available resources	(6) The weekly personal contact with the professional during the program made me feel assisted and welcomed. (9) I would not go back to doing online activities with this professional or with this team. (11) The online exercise program positively interfered with my family/friend's relationship.	These questions focused on the quality of content, enthusiasm while practicing the exercises, the effects on general health, and comparison between the remote and the face-to-face program.
Caregiver or family member	Family member's or caregiver's perceptions related to the program	(13) My family member felt comfortable performing physical exercises online with my support beside him (her). (14) According to the instructions, I received from the team members each session, it was possible to help my family member to carry out the exercises. (15) Helping my family member during the program interfered negatively with my routine, because it took a lot of time. (16) Monitoring my family member was easy and did not physically require much effort on my part. (17) I considered my family member and I had fun doing physical exercises every session. (18) I think the online physical exercise program positively interfered with my family's life during the COVID-19 pandemic period.	These questions addressed the family member or caregiver's perception about the remote program, the need for support and attention from the participants.

Once a month, the researcher team inquired the participants and their family members or caregivers about the overall experience (i.e., motivation, involvement, understanding of activities, connectivity, communication, interaction, satisfaction, and quality of life regarding both professionals and exercise sessions) using an 18-item questionnaire created by the authors. Participants answered 12 questions, while family members/caregivers answered the other 6 questions. After the phone call with the participant, the call was directed to the caregiver, who answered the corresponding questions in the questionnaire. The same caregiver answered these questions every month. A 5-point Likert scale was applied based on the positive and negative statements (Likert, 1932). The answers were followed at five levels; for positive statements, “fully disagree” was 1 and “fully agree” was 5, while for negative statements, “fully disagree” was 5 and “fully agree” was 1. The average time to complete the questionnaire was 20 min. The maximum score was 55 points and the minimum score was 11 points. The positive questions include 1, 2 5, 6, 7, 8, and 11. Question 12 was used to classify whether the participants needed a family member or caregiver to perform the sessions. Regarding the family members or caregiver questionnaire, questions 13, 14, 15, 16, and 18 were positive. The minimum score was 6 points and the maximum score was 30 points (Interactive Presentation Software–Mentimeter, 2020).

### Statistical Analysis

Mean, mode, and median were used to depict data information. SD and 95% CI were used to describe data variability. We used Origin (version 2020, Origin Lab Corp, Northampton, MA, USA) for the plots and SPSS (version 20, IBM Corp, Armonk, New York, NY, USA) for statistical analysis.

The adherence rate was defined as the relative frequency of individuals who accepted to engage in the remote exercise program (Ellis et al., 2019; Landers and Ellis, 2020). The attendance rate was calculated by the median of the number of performed sessions (based on the total of 48 sessions) and the mean of accumulated time per week of exercise performed (in minutes).

Furthermore, we aimed to describe the higher and lower frequencies of attendance, including the participants who attended more than 80% and <20% of the sessions. The barriers of attendance were described as the frequency based on the weekly report. Safety was described as the frequency of reported adverse effects per session. The overall experience was described as the median and range of the monthly questionnaire responses.

For the overall experience questionnaire, median, first and third quartiles, and range were presented for each question.

## Results

Table 3 describes the sample characterization, demographic, and assessment data referring to the initial evaluation. The sample was composed of individuals in the chronic phase of the stroke, who were community walkers based on the 10 MWT. Their aerobic capacity was categorized as an unlimited walker based on the 6 MWT. In terms of risk of falls, they present a low risk that was assessed by the TUG and a moderate risk of falling that was assessed by the Mini-BESTest. The confidence to do activities that require balance was well-assessed by ABC. Cognition, according to MoCA criteria, shows that most of the participants had cognitive impairment. The perception of the participants about the quality of life was good with a high report of the feeling of recovery after the stroke, according to SIS.

**Table 3:** Demographic sample characterization.

**Variable**	**Mean (Standard deviation)**
Sex^a^	23 M/17 F
Age (year)	55.1 (15.3)
Schooling^b^ (year)	10.6 (5.1)
Time since stroke (month)	98 (63.8)
Type of stroke^a^	26 I/11 H/3 both
Affected hemisphere^a^	13 R/20 L/4 both
10-m walk test (m/s)	0.9 (0.3)
TUG test (s)	14.7 (9.1)
6-min walk test (min)	302.9 (105.5)
MiniBESTest	19.9 (5.5)
ABC (%)	74 (16)
MoCA test	21.6 (4.5)
SIS	222.5 (28.6)
SIS (%)	75 (18)

a *Simple count; M, male; F, female*;

b *Years studied, I, ischemic; H, hemorrhagic; R, right; L, left; TUG, Time up and Go test; MiniBESTest, Mini-Balance Evaluation Systems test; ABC, activity-specific balance confidence scale; MoCA, Montreal Cognitive Assessment; SIS, Stroke Impact Scale*.

### Adherence

**Figure 2:**
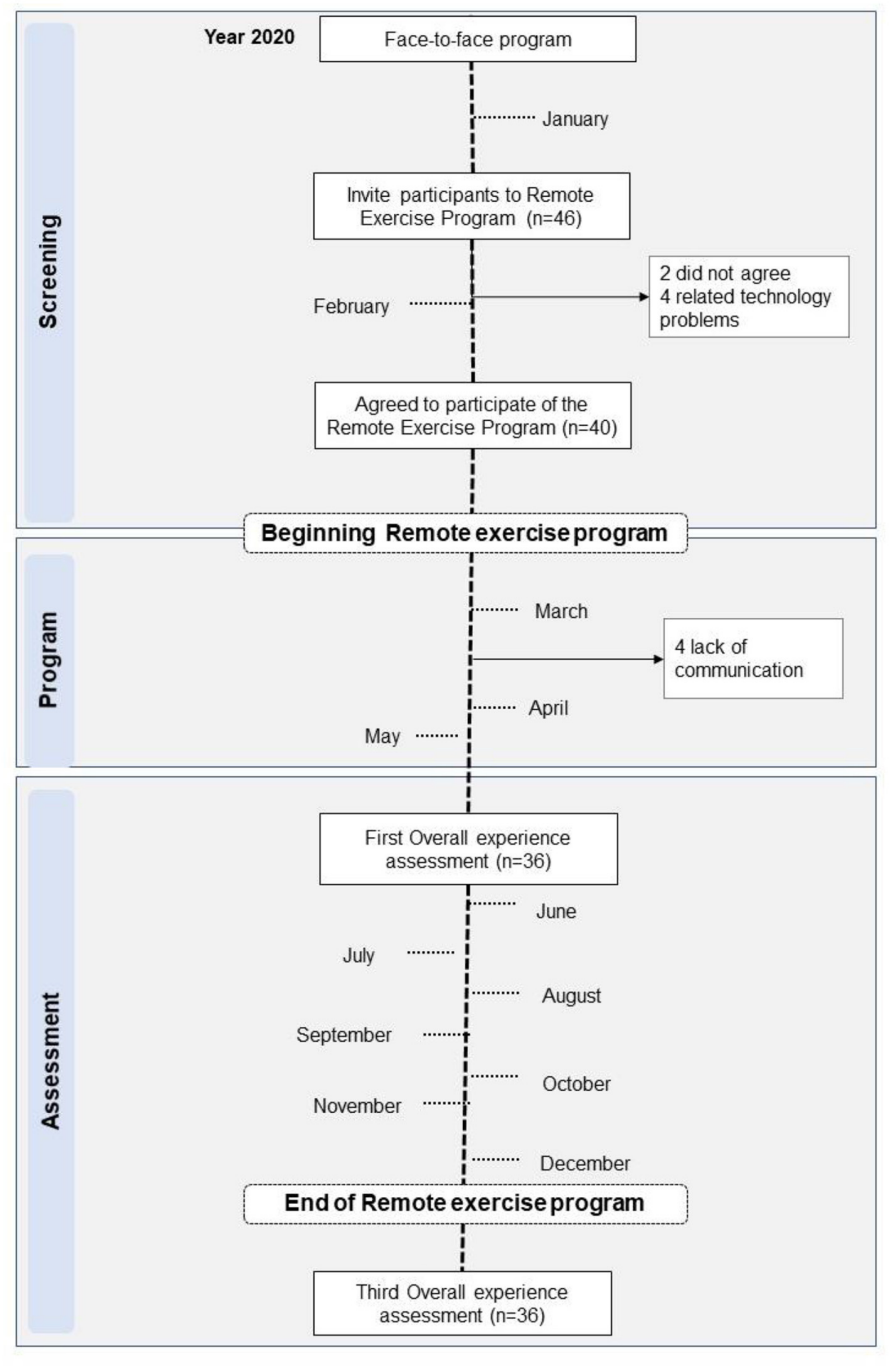
Flowchart of the study.

The study flowchart is described in Figure 2. We have invited 46 individuals who were engaged in a face-to-face physical exercise program for stroke survivors. Two participants who were invited did not want to attend the remote program, and another four were excluded due to technological problems. Forty participants have accepted and engaged in the remote exercise program. The adherence to the program was 86.9%. However, during this study, three participants had problems with communication and were not available for the weekly phone calls and one of them had a medical problem. Therefore, 36 of them participated in the final overall experience.

#### Attendance

##### Sessions

A total of 48 sessions, for a duration of 24 weeks, were delivered through the asynchronous remote exercise program. Average individual attendance was 19.8 ± 14.8 sessions (44.9 ± 33.7%, 95% CI 15.2–24.4). Ten (25%) participants attended 80% or more sessions, and 13 (32.5%) participants attended <20% of the sessions.

##### Volume

Based on the sessions attended, the active participants utilized 99.1 ± 19.4 min/week of physical exercise of the program.

#### Barriers

Pandemic refers to the changes in the daily routine due to the home office and the need to move out with the family to assure social isolation. The health-related factors include lack of physical capacity, impairments due to the stroke, and other health problems that may have been described as barriers to performing the remote physical exercise program (Table 4).

**Table 4:** Absolute and relative frequency of reported barriers.

**Barriers**	**Definition**	***N***	**%**
**Health condition-related barriers**			
Lack of motor skills and physical fitness to workout	Muscle weakness, lack of balance, lack of motor coordination, mobility.	80	20.6
Health condition appointments	Medical appointments, medication effects, injury recovery, dizziness, labyrinthitis, seizure.	37	9.5
Constrains to use the most affected side while exercising	Partial or total paralysis on one side of the body affecting the capability to perform the video exercises prescribed.	31	8.0
Lack of time	Lack of routine organization and time.	30	7.7
Pain	Presence of pain (e.g., shoulder, lumbar, spine, knee, leg).	28	7.2
Behavioral issues	Lack of motivation and tiredness.	17	4.4
Constraints to do mat workout	Difficulty performing the physical exercises on the mat due to lack of mobility.	15	3.9
Fear of injury	Feeling insecure to perform the exercise.	9	2.3
Personal issues	Personal reasons not specified.	8	2.1
Fear of fall	Fear of fall while performing the exercise.	3	0.8
Dual-Task performance	The participant feels he (she) is unable to perform two tasks simultaneously.	1	0.3
Grieve	Emotional issues related to the loss of a family member.	1	0.3
**Environmental related barriers**			
No exercise companions	The caregiver or family member could not help the participant, lack of motivation to exercise due to being alone.	44	11.3
Problems with communication and lack of knowledge to use internet devices and tools	Problems with internet connection and the use of mobile phone difficulty to access or download video sessions.	21	5.4
Lack of a safe place to exercise	Lack of adequate space to exercise.	8	2.1
Domestic life	Daily life commitments (e.g., homeschooling, house repairs, relatives' appointments).	7	1.8
Weather condition	Climate conditions related to the seasons of the year (cold or hot weather).	3	0.8
**Pandemic related barriers**			
Job commitments during the coronavirus pandemic	Due to the pandemic, individuals do not have time to do the exercises because they have to do the home office.	17	4.4
Coronavirus pandemic issues	The caregiver or family member does not have time to help (routine changed dramatically due to pandemic); the family member or caregiver was diagnosed with Covid-19.	16	4.1
Traveling	The participant had to travel with the family and was unable to access the video sessions.	12	3.0
Total			100%

To illustrate the most cited health conditions-related barrier (lack of motor skills and physical fitness to workout−20.6%), we described the following statements:

“*I have difficulty to perform the exercises using my affected upper limb. Although I see the staff member performing the exercise on the video, I cannot do the same. I have difficulty going to the mat and exercise on the floor. I feel I cannot stand up safely by myself. It is difficult to stand up.”*

To illustrate the environmental-related barriers, we have extracted sentences from the reports of the participants:

“*I don't have a companion to exercise with myself because my daughter says she does not have time to assist me during the activities due to her professional commitments.”*

Other aspects that were included as environmental-related barriers were “problems with communication and lack of knowledge to use Internet devices and tools” (5.4%). To illustrate this barrier, we extracted the following sentence:

“*I have difficulty accessing the videos because of poor Internet connection.”*

The main barrier related to the pandemic construct was “job commitments during the coronavirus pandemic.” To illustrate it, we presented the following statement from the participants:

“*I can't manage my work hours with my need for physical exercise. It seems even harder to do home office during this pandemic situation.”*

#### Safety

In 48 sessions, the individuals described 45 reports (5.7%) of pain during the sessions and pain a day after the session in 50 reports (6.3%). Participants just reported one fall (0.1%), dizziness in 30 reports (3.8%), motion sickness in seven reports (0.9%), and fear of exercising in 92 reports (11.6%) during the exercise sessions. In terms of activity, for every 10,000 min, there was 0.2% fall, 9.5% pain reports during the session, 10.5% pain reports a day after the session, 6.3% seizures, 1.5% motion sickness, and 19.4% fear of exercising reports during the sessions.

### Overall Experience

For the personnel construct, the median answer for question 2 was 5 (range 3), and for question 3 the median was 1 (range 4). In question 2, participants agreed with “to feel safe in activities.” In question 3, participants strongly disagreed with the fact that the program interfered negatively with their mood.

For the communication construct, the median answer to question 7 was 5 (range 4) and question 10 was 1 (range 4); therefore, participants fully agreed with how easy the communication with the team members was established, and question 10 reflects that they had problems with technology and the Internet connection.

For the program construct, the median answer for questions 1 and 8 was 5, but with different ranges (3 for question 1 and 4 for question 8), question 4 was 3 (range 4), and question 5 was 1 (range 4). Thus, participants felt involved (question 1) and supported (question 8) with the program, but neither they felt that they were able to perform all the proposed activities (question 4), nor they think that the remote program should replace the face-to-face program.

For the social support construct, the median answer for questions 6 and 11 was 5 (range 4), and question 9 was 1 (range 4); thus, participants felt assisted (questions 6), which positively affected their relationships (question 11), and they disagreed about the fact that they would not do activities with these professionals or teams again (question 9).

For the caregiver construct, the median answer for questions 13 and 14 was 5 (ranges 3 and 4), question 15 was 1 (range 1), question 16 was 4 (range 4), and questions 17 and 18 were 5 (ranges 2 and 4). Thus, the family member or caregiver felt comfortable doing exercises with their family members (question 13), and he/she was assisted by the instructions they had (question 14). The family members and caregivers did not feel affected in their routine by supporting the participant (question 15). They thought that monitoring family members did not require much effort (question 16). They had fun doing the exercises with their family member involved in the remote program (question 17) and the overall experience doing the exercises positively affected their life during the pandemic (Table 5).

**Table 5:** Median and range results across the answers of all the participants for the overall experience questionnaire.

**Domain**	**Questions**	**Results**
Personnel	(2) The program made me feel safer in activities at home, such as walking. (3) The program interfered negatively with my mood and general health.	2–“I fully agree,” **Median 5 (range 4)** 3–“I fully disagree,” **Median 1 (range 4)**
Communication	(7) During the online exercise program, I had easy communication with the professional team. (10) During the online exercise program, I had difficulties connecting with technology and I had problems with my internet connection.	7–“I fully agree,” **Median 5 (range 1)** 10–“I fully disagree,” **Median 1 (range 4)**
Program	(1) The format of this online program allowed me to participate and get involved satisfactorily. (4) I was not able to carry out all the activities and exercises prescribed to me. (5) I liked the online program and I think it could replace the face-to-face program. (8) During sessions, the professional showed that he/she understood my difficulties and managed to feedback on my performance and provide the needed support to me.	1–“I fully agree,” **Median 5 (range 1)** 4–“Neutral,” **Median 3 (range 4)** 5–“Fully disagree,” **Median 1 (range 4)** 8–“Fully agree,” **Median 5 (range 4)**
Social support	(6) The weekly personal contact with the professional during the program made me feel assisted and welcomed. (9) I would not go back to doing online activities with this professional or with this team. (11) The online exercise program positively interfered with my family/friend's relationship.	6–“I fully agree,” **Median 5 (range 4)** 9–“I fully disagree,” **Median 1 (range 4)** 11–“I fully agreed,” **Median 5 (range, 4)**
Caregiver or family member	(13) My family member felt comfortable performing physical exercises online with my support beside him (her). (14) According to the instructions, I received from the team members each session, it was possible to help my family member to carry out the exercises. (15) Helping my family member during the program interfered negatively with my routine, because it took a lot of time. (16) Monitoring my family member was easy and did not physically require much effort on my part. (17) I considered my family member and I had fun doing physical exercises every session. (18) I think the online physical exercise program positively interfered with my family's life during the COVID-19 pandemic period.	13–“I fully agree,” **Median 5 (range 1)** 14–“I fully agree,” **Median 5 (range 3)** 15–“I fully disagree,” **Median 1 (range 4)** 16–“I agree,” **Median 4 (range 4)** 17–“I fully agree,” **Median 5 (range 2)** 18–“I fully agree,” **Median 5 (range 4)**

*Median of responses of questionary and range (ranking) of these responses are indicated in bold*.

## Discussion

This study has evaluated adherence, attendance rate, and barriers to attend to a remote physical exercise program for stroke survivors. Our remote physical exercise program presented a high adherence. The barriers to attending the remote exercise program were difficulties related to health conditions (including the use of the most affected side), the pandemic (change in daily routine and implications imposed by social isolation), and environmental issues (lack of company to perform the exercises, or problems with family members or caregivers). These barriers were different from other studies with the same population. In general, barriers to exercise are described to understand why sedentary people do not engage in physical exercise activities. Due to the COVID-19 pandemic, barriers to switching between a face-to-face group exercise program to a remote mode of delivery are mandatory. Our results show that specific barriers have emerged from this condition.

The adherence to the remote physical exercise program was higher compared with the literature (Beit Yosef et al., 2019; Wu et al., 2020). Such a high adherence rate might have two reasons, namely, these participants were already engaged in a face-to-face physical exercise and they were highly informed about how important it is to exercise. The two recommendations from WHO for a better life, namely, health education and an active lifestyle, are the aims of our face-to-face and remote programs (van Wijck et al., 2019).

While two participants who were involved in the face-to-face exercise program refused to engage in the remote program, four other participants who reported difficulties with Internet connectivity have also refused to join the remote program. In Brazil, the stroke prevalence is higher in individuals with low education and low income (O'Donnell et al., 2016; De Santana et al., 2018). Although only 8.7% participants involved in the face-to-face program have reported poor Internet connection to not engage, such results suggest that the low-income population also has reasonable Internet connection or such conditions (low education and low income) are not common features in our group.

Mean participation was less than half of all sessions, impacting the attendance rate. Only 10 participants have attended more than 80% of the sessions. This finding suggests how important it is to motivate and tailor the remote physical exercise program to the need of each stroke survivor. Our participants have different clinical impairments, and they might have not felt safe and comfortable to exercise. Studies (van Wijck et al., 2019; Wu et al., 2020) suggest that the need to attend a remote program is related to transportation issues (home-to-facility distance or access to reference rehabilitation centers). Although they have approved the remote program, they did not agree to stay on the remote mode when the face-to-face mode was available again (Chen et al., 2020).

To attend a remote physical exercise program during the COVID-19 pandemic is a human development issue (that involves health, education, and economics). Our results must be read through the cultural lenses of a country, highly impacted by the COVID-19 socially and economically (Tyagi et al., 2018). The need for a family member or a caregiver to support the attendance of the participants and the basic education level of the participants are factors that affect the motivation to engage in a remote physical exercise program. A stimulating environment can promote an active life and counteract these factors. On a synchronous mode, a remote physical exercise would increase social bonding with other participants and could improve motivation and self-efficacy (Lewthwaite and Wulf, 2012; Wulf and Lewthwaite, 2016).

This remote physical exercise program was important to reach WHO recommendations for a healthy life. Active participants covered about two-thirds of WHO recommendations (150 min of moderate activity per week) (Bull et al., 2020). Information concerning the time spent in moderate to vigorous physical activity for stroke survivors is scarce (Fini et al., 2017). For the subacute phase, stroke survivors did, on average, 27 min/day. It is a study limitation not to control how intense were the exercises. For fragile populations, reaching vigorous intensity in exercising can be a high-risk activity without the proper support, which is barely available at home. Participation in our study was an important action to make our attendees physically active.

The main barrier to attending the remote program was the difficulty to do the proposed exercises due to motor impairments. To tailor exercises to personal features was a challenge at this remote model because each participant had different impairments. The asynchronous participation gives opportunities to the attendees to judge how safe it is to do the whole exercise session and also demands self-motivation to engage, family support, and the willingness for a healthy lifestyle after a stroke.

The second most related barrier was the lack of someone to aid the exercise at home. Even though they are considered independent for self-care and walking, stroke survivors have limitations of activities, such as physical exercise (Campos et al., 2019). Despite the physical exercise being carried out at home, he/she depended on a caregiver to ensure the organization of the environment and the safety in the execution of the exercises. During our orientation, safety for carrying out the exercises was emphasized to minimize adverse effects.

In the category of pandemic issues, “job commitments during the pandemic coronavirus” was the most frequent issue. This finding correlates with the present environmental category, a profile of individuals who present a perception that their physical condition is a barrier to physical exercise and they need a family member or caregiver to advise them. However, those family members/caregivers are facing new home office obligations due to the change in routine caused by the COVID-19 pandemic.

Most of the participants reported that the remote physical exercise was safe. Just one fall was reported across all the sessions. To the best of our knowledge, this is the first telemonitoring study for stroke survivors to report these data. Individuals tend to experience social discomfort, loss of confidence and personal identity, long-term functional disability, and loss of independence when falls happen (Schmid and Rittman, 2009). To manage falls and their consequences is fundamental to improve self-efficacy and autonomy. Our video sessions contained safety instructions, and all participants were encouraged to pay attention to this issue when performing the exercises. Rare adverse events were reported in our study suggesting that remote exercises program are safe for stroke survivors (Piotrowicz et al., 2015).

Pain complaint was one of the most reported issues. The main changes related to physical activity are a high frequency of pain and discomfort (Jones et al., 2016). Participants expressed such feelings:

“Physical activity causes me pain or discomfort.”

“I think physical activity is very tiring.”

“I am afraid to do my physical exercises alone.”

Opinions of the participants concerning the overall experience about this remote program suggest that it was safe from the view of the participants, thus allowing a major interaction with the program. The ease of communication between the participants and the team allowed us to be aware of all the issues that harmed the progress of the program and allowed us to correct these flaws.

According to reports of the program, it was well-accepted, despite the fact that most of the participants prefer the face-to-face program. Remote programs are well-accepted (Chen et al., 2017; Sarfo et al., 2018), and this acceptability impacts the social support related to them in a positive way. The recognition and the knowledge of the points of view of the participants are important and are currently missing in many studies (Laver et al., 2020). The family members or caregivers also had a good experience overall with this remote physical activity program. Our results express the presence of a family member as a facilitator for the commitment of remote physical exercise (Chen et al., 2017; Sarfo et al., 2018).

Due to the COVID-19 pandemic, our sample was selected by convenience and all participants were already engaged in a face-to-face program. Another limitation was not having a control group with no intervention or face-to-face intervention to contrast our results. Our questionnaires were not previously validated but were developed for this study. It would be important to add other tools to evaluate the psychosocial domains within a telemonitoring program.

## Conclusions

The remote exercise program has a high adherence rate among stroke survivors to engage and a low attendance rate. The main reported barriers involved the need to individually tailor the exercises to personal impairments. Attending a remote physical exercise program is safe, and the overall experiences of the participants and their family members/caregivers were positive. A remote physical exercise program can reduce sedentary behavior and increase the physical activity level in individuals who had a stroke.

## Data Availability Statement

The original contributions presented in the study are included in the article/supplementary material, further inquiries can be directed to the corresponding author/s.

## Ethics Statement

The studies involving human participants were reviewed and approved by School of Physical Education and Sport of the University of São Paulo, São Paulo, Brazil. The patients/participants provided their written informed consent to participate in this study.

## Author Contributions

CT-P: an idealizer and coordinator of the physical activity program, coordinated the conversion of the face-to-face model to the remote program, collaborated in the creation of the questionnaires, coordinated the collections, and writing and revision of the text. GP, MM, and BA: conversion of the face-to-face model to the remote program, collaborated in the creation of the questionnaires, monitored the individuals, and writing and revision of the text. AL: creation of the questionnaires and writing and revision of the text. TS, MC, and RJ: performed individuals monitoring and data analysis. VD and TF: conversion face-to-face model to the remote program and collaborated in the creation of the questionnaires. LM: creator of the project, creation of the questionnaires, performed data analysis, text writing, and text review. All authors contributed to the article and approved the submitted version.

## Conflict of Interest

The authors declare that the research was conducted in the absence of any commercial or financial relationships that could be construed as a potential conflict of interest.
